# The Type III Intermediate Filament Protein Peripherin Regulates Lysosomal Degradation Activity and Autophagy

**DOI:** 10.3390/ijms26020549

**Published:** 2025-01-10

**Authors:** Roberta Romano, Paola Cordella, Cecilia Bucci

**Affiliations:** Department of Experimental Medicine, University of Salento, Via Provinciale Lecce-Monteroni n. 165, 73100 Lecce, Italy; roberta.romano@unisalento.it (R.R.); paola.cordella@unisalento.it (P.C.)

**Keywords:** peripherin, intermediate filaments, cytoskeleton, lysosome, autophagy

## Abstract

Peripherin belongs to heterogeneous class III of intermediate filaments, and it is the only intermediate filament protein selectively expressed in the neurons of the peripheral nervous system. It has been previously discovered that peripherin interacts with proteins important for the endo-lysosomal system and for the transport to late endosomes and lysosomes, such as RAB7A and AP-3, although little is known about its role in the endocytic pathway. Here, we show that peripherin silencing affects lysosomal abundance but also positioning, causing the redistribution of lysosomes from the perinuclear area to the cell periphery. Moreover, peripherin silencing affects lysosomal activity, inhibiting EGFR degradation and the degradation of a fluorogenic substrate for proteases. Furthermore, we demonstrate that peripherin silencing affects lysosomal biogenesis by reducing the TFEB and TFE3 contents. Finally, in peripherin-depleted cells, the autophagic flux is strongly inhibited. Therefore, these data indicate that peripherin has an important role in regulating lysosomal biogenesis, and positioning and functions of lysosomes, affecting both the endocytic and autophagic pathways. Considering that peripherin is the most abundant intermediate filament protein of peripheral neurons, its dysregulation, affecting its functions, could be involved in the onset of several neurodegenerative diseases of the peripheral nervous system characterized by alterations in the endocytic and/or autophagic pathways.

## 1. Introduction

Intermediate filaments (IFs), together with microtubules and microfilaments, are a component of the cytoskeleton, an extremely dynamic structure involved in several cellular processes such as cell division, intra-cellular transport, motility, reaction to external forces, adhesion, and the adaptation of cell shape [1,2].

The cytoskeleton is able to organize the inside of the cell, to connect cells physically and biochemically to the extracellular environment, to generate coordinated forces necessary to cell movements, and to change cell shape. All cytoskeletal structures are organized into networks that can reorganize quickly in response to external forces, maintaining the integrity of the intracellular environment, controlling the cell shape, and organizing cellular components. The activity of numerous cytoplasmic proteins and organelles is integrated by the cytoskeleton, which should not be viewed as a fixed and isolated structure but as a highly dynamic structure whose components are in constant flux [3]. The networks formed by the three cytoskeletal polymers can be distinguished by their stiffness, the mechanism of their assembly, their polarity, and the type of molecular motors associated with them.

The stiffest polymers are represented by microtubules which are also characterized by the most complex assembly and disassembly mechanisms [4]. Less rigidity is shown by actin filaments, but the activity of a lot of crosslinkers binding actin generates stiff structures such as bundled or branched networks. The former are involved in chemotaxis and cell–cell communication, while the latter support the leading edge of motile cells and generate the forces necessary to change the cell shape, for example, during phagocytosis [5]. Actin filaments and microtubules are polarized polymers, and they function as tracks for molecular motors, moving preferentially in one direction (myosin proteins for microfilaments and dynein and kinesis families for microtubules) [3]. Less rigidity characterizes Ifs, which are more effective in resisting tensile forces rather than compressive forces. They can crosslink to each other, and some IFs can also interact with microtubules or actin filaments. Unlike microtubules and microfilaments, IFs are not polarized [6].

IFs represent the most heterogeneous class, which comprises proteins encoded by at least 70 genes [7,8,9]. According to their structure and sequence, IFs are grouped into five classes. Classes I-IV contain cytoplasmic IFs, while class V contains lamins, the only nuclear IF proteins. Filensin and phakinin are two IF proteins not grouped into the five mentioned classes [1]. All of these proteins share a common structure represented by two non-α-helical domains separated by a central α-helical rod domain [10]. According to in silico structural prediction, in the central region, amino acids are organized in heptad repeats, which form four sub-helices (coil 1A, coil 1B, coil 2A, and coil 2B) that are separated by three linker domains (L1, L12, and L2) [11,12]. Post-translational modifications, such as phosphorylation and acetylation, regulate the organization and function of Ifs [13,14].

IFs form a dense network mainly located in the perinuclear space, but they also extend to the cortex, where they can interact with desmosomes and hemidesmosomes to maintain cell adhesion [15,16,17,18]. IFs form a scaffold for mitochondria, with the Golgi apparatus and other organelles being important to their location [19,20,21]. Thanks to their mechanical properties, IFs maintain cell and tissue integrity, but in addition to this “traditional” function, they also have important roles in apoptosis, migration, adhesion, and vesicular trafficking [1,22]. Regarding the latter function, evidence for IF involvement in the endo-lysosomal pathway was derived from the discovery of the interaction between the IF proteins vimentin, peripherin, and α-internexin with the adaptor protein AP-3 (adaptor protein complex 3), which is involved in the sorting of proteins in the endo-lysosomal system [23].

Other evidence of the involvement of IF proteins in the endo-lysosomal system came from the discovery that keratin 8 is important for the formation of autophagosomes [24]; GFAP (glial fibrillary acidic protein), and vimentin for the mobility of endosomes and lysosomes [25,26]; neurofilament light (NfL) for lysosomal transport [27]; vimentin for the positioning of late endosomal–lysosomal compartments, the luminal ionic composition of endocytic organelles, and the content of autophagosomes [23]. Therefore, several studies demonstrated how IFs are important for the regulation of lysosomal cargoes and the luminal content of late endosomes and lysosomes, for the distribution and motility of late endocytic compartments, and for autophagy, highlighting the strong connection between IFs and vesicular trafficking [7].

Little is known about peripherin, an IF protein belonging to the heterogeneous class III together with vimentin, desmin, and GFAP. Peripherin is the only IF protein selectively expressed in neurons of the peripheral nervous system (PNS) and in neurons of the central nervous system that project toward peripheral neurons, such as spinal cord motoneurons [28,29,30,31]. It has been demonstrated that peripherin is involved in fast axonal transport [27] and neurite growth and stability [32]. Indeed, pheochromocytoma PC12 cells lose their ability to form neurites upon peripherin silencing, remaining morphologically identical to undifferentiated cells [33]. We have demonstrated that, in addition to AP-3, peripherin also interacts with RAB7A (Ras-related in brain 7A) [34], a small GTPase of the RAB family which is involved in the transport to late endosomes and lysosomes and in the biogenesis of lysosomes, phagolysosomes, and autolysosomes [35,36,37]. RAB7A is able to regulate peripherin assembly as RAB7A silencing or overexpression alters the soluble/insoluble ratio of peripherin [34].

Peripherin is a marker for neuronal injury and degeneration [38,39,40,41], but its specific functions in cells remain to be discovered. Indeed, there is no direct evidence of its role in vesicular trafficking and, to date, its involvement can only be hypothesized based on its interaction with proteins important for the endocytic pathway and on its homology with other IFs belonging to class III, such as vimentin. Therefore, the aim of this work was to investigate the role of peripherin in the endocytic and autophagic pathways. We demonstrated that peripherin is important for the correct positioning and functioning of lysosomes, thus affecting the endocytic and autophagic pathways.

## 2. Results

### 2.1. Peripherin Silencing Affects LAMP1 Abundance and Lysosomal Positioning

**Figure 1:**
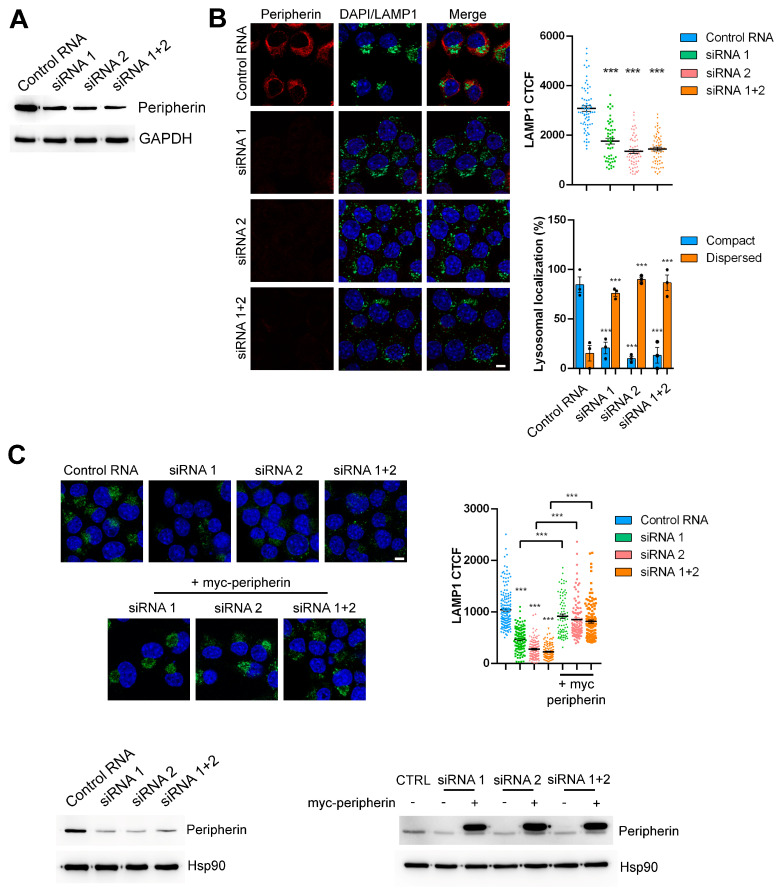
Peripherin silencing affects LAMP1 abundance and lysosomal localization. (**A**,**B**) Neuro2a cells were treated with 2 siRNAs against peripherin mRNA, individually or in combination, or with a control RNA as a negative control. After 96 h of transfection, the cells were lysed, and the samples were subjected to a Western blot analysis using antibodies against peripherin with GAPDH used as the loading control (**A**), or cells were fixed, permeabilized, and immunolabeled with anti-peripherin and anti-LAMP1 antibodies followed by Alexa568- and Alexa488-conjugated secondary antibodies, while the nuclei were stained with DAPI (**B**). Bar = 10 µM. ImageJ software 1.54d was used for the CTCF calculation, and the percentage of cells with compact or dispersed lysosomes was calculated. (**C**) Neuro2a cells were silenced as indicated for 48 h and then transfected for 48 h with a plasmid encoding myc-tagged peripherin. Cells were then fixed, permeabilized, and immunolabeled with anti-LAMP1 antibody followed by an Alexa488-conjugated secondary antibody. Nuclei were stained with DAPI. Bar = 10 µM. The CTCF was calculated using ImageJ. To control silencing and transfection, a Western blot analysis was performed using antibodies against peripherin, and Hsp90 was used as the loading control. *** = *p* < 0.001.

In order to investigate the role of peripherin on lysosomal morphology, positioning, and functioning, we decided to silence the peripherin gene using RNA interference. To perform our experiments, we used the Neuro2A mouse neuroblastoma cell line known to express peripherin [34]. In Neuro2A cells, we tested two different siRNAs designed against the mouse peripherin mRNA, and we used them individually or in combination. To analyze their efficacy in silencing, we looked at the amount of the peripherin protein using a Western blot analysis (Figure 1A). Both siRNAs were able to induce good silencing of peripherin and, notably, when cells were treated with the two siRNAs together, silencing efficiency was even higher (Figure 1A). For this reason, a number of the experiments presented in this paper have been performed with a combination of the two siRNAs.

In order to assess the effect of peripherin silencing on lysosomes, we silenced Neuro2A cells with the two siRNAs, individually or in combination, and we evaluated the abundance and distribution of LAMP1 (lysosomal associated membrane protein 1), a marker of late endosomes and lysosomes [42,43], using confocal microscopy. Interestingly, peripherin silencing caused a decreased abundance of LAMP1 staining, as demonstrated by the statistically significant reduction in Corrected Total Cell Fluorescence (CTCF) (Figure 1B). Moreover, in control cells, LAMP1 staining was mainly localized in organelles clustering in the perinuclear area, as was previously demonstrated for late endosomes and lysosomes. However, in peripherin-silenced cells, staining was more dispersed in the cytoplasm, and LAMP1-positive organelles reached the cell periphery (Figure 1B). These data suggest that peripherin silencing affects not only the number of lysosomes present in the cells but, importantly, also the positioning of these organelles, as in silenced cells, the late endosomes and lysosomes labeled by LAMP1 are not clustered in the perinuclear region anymore.

Having demonstrated that peripherin silencing with the two siRNAs, individually or in combination, affects lysosomal abundance and positioning, we wondered if the re-expression of peripherin in silenced cells was able to rescue LAMP1 abundance. Interestingly, when peripherin was re-expressed in silenced cells, LAMP1 abundance increased to levels comparable to control cells (Figure 1C). Furthermore, the clustering of late endosomes and lysosomes in the perinuclear area was restored (Figure 1C). Altogether, these data indicate that the observed phenotype was determined directly by peripherin silencing, and it was not due to the off-target effects of the siRNAs (Figure 1C).

### 2.2. Peripherin Silencing Impairs Lysosomal Functionality and Autophagy

**Figure 2:**
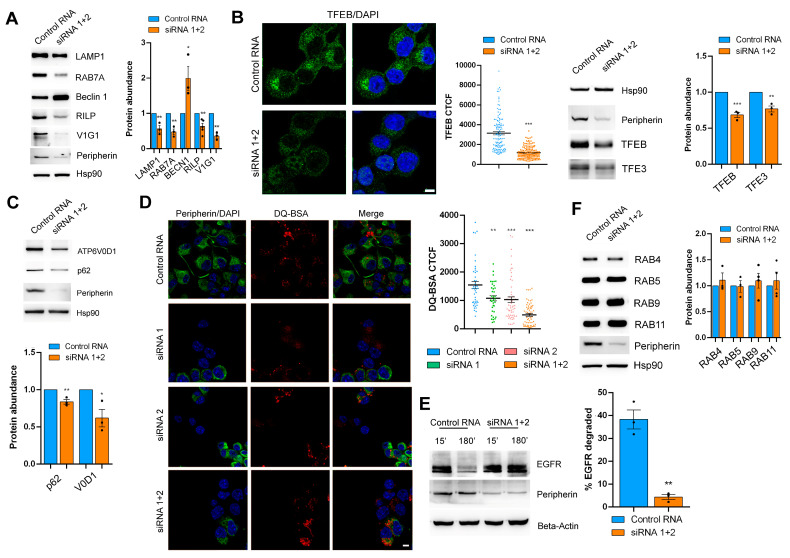
Peripherin silencing affects lysosomal functionality. (**A**) Lysates of peripherin-silenced or control Neuro2a cells, as indicated, were subjected to Western blot analysis using antibodies against LAMP1, RAB7A, beclin 1, RILP, V1G1, peripherin, and Hsp90. Relative protein abundance was quantified by densitometric analysis normalizing against Hsp90. (**B**) Peripherin-silenced or control Neuro2A cells, as indicated, were subjected to immunofluorescence analysis using anti-TFEB antibody followed by Alexa488-conjugated secondary antibody, while nuclei were stained with DAPI. Bar = 10 µM. TFEB CTCF was calculated using ImageJ software. To check silencing efficiency, Western blot analysis was performed on same samples using antibodies against peripherin and Hsp90 as loading control. Samples of control and silenced cells were also subjected to Western blot analysis using antibodies against TFEB and TFE3. Relative protein abundance was quantified by densitometric analysis normalizing against Hsp90. (**C**) Lysates of control or peripherin-depleted Neuro2a cells were subjected to Western blot analysis using antibodies against RAB4, RAB5, RAB9, RAB11, peripherin, or Hsp90 as loading control. Relative protein abundance was quantified by densitometric analysis normalizing against Hsp90. (**D**) Peripherin-silenced or control Neuro2A cells were incubated with DQ-Red BSA for 8 h and then fixed and immunolabeled using anti-peripherin antibody followed by Alexa488-conjugated secondary antibody. Nuclei were stained with DAPI. Bar = 10 µM. DQ-BSA CTCF was calculated using ImageJ. (**E**) Peripherin-silenced or control Neuro2A cells were treated with cycloheximide for 1 h and then stimulated with EGF for 15 min and 3 h. Lysates were then subjected to Western blot analysis using antibodies against EGFR, peripherin, and β-actin as loading control. Percentage of degraded EGFR was calculated. (**F**) Lysates of control or peripherin-silenced Neuro2a cells were subjected to Western blot analysis using antibodies against p62, V0D1, peripherin, or Hsp90 as loading control. Relative protein abundance was quantified by densitometric analysis normalizing against Hsp90. * = *p* < 0.05; ** = *p* < 0.01; *** = *p* < 0.001.

Having demonstrated that peripherin silencing affects LAMP1 abundance by confocal microscopy, we next investigated the expression of other proteins important for the autophagy–lysosomal pathway (ALP) using a Western blot analysis. We confirmed that the expression of LAMP1 was reduced upon peripherin silencing (Figure 2A). Also, the expression of RAB7A (a small GTPase that controls the late endocytic pathway and the autophagic flux), RILP (Rab-Interacting Lysosomal Protein, a RAB7A interactor which recruits the dynein–dynactin motors promoting the transport of organelles toward the minus-end of microtubules), and V1G1 (a subunit of the V-ATPase whose abundance is regulated by RILP) was reduced in peripherin-silenced cells (Figure 2A). In contrast, Beclin 1 (a key factor involved in autophagosome biogenesis) was more abundant [35,44,45,46,47,48,49] (Figure 2A).

To further investigate the influence of peripherin silencing on ALP markers, we decided to look at TFEB (transcription factor EB), which plays a pivotal role in lysosomal biogenesis and autophagy [50,51]. In immunofluorescence experiments, we observed predominant cytoplasmic staining of TFEB according to the nutrient-rich conditions in which cells have been cultivated. The presence of nutrients causes TFEB retention in the cytoplasm since its nuclear localization signal is masked by 14-3-3 proteins following TFEB phosphorylation by mTOR (mammalian target of rapamycin), determining its larger localization in the cytosol [52,53,54,55,56].

However, when we looked at the total TFEB levels, we observed that TFEB abundance was negatively influenced by peripherin silencing (Figure 2B), suggesting the impairment of lysosomal biogenesis. To confirm this finding, we looked at another master regulator of lysosomal biogenesis, TFE3 (transcription factor E3), and we observed its downregulation in peripherin-depleted cells as well (Figure 2B). To further confirm that lysosomal biogenesis was affected by TFEB downregulation, we analyzed the protein products of two TFEB target genes, the V-ATPase (vacuolar ATP-ase) subunit ATP6V0D1 and p62/SQSTM1 (sequestosome 1). Interestingly, both were downregulated following peripherin silencing (Figure 2C).

Then, we evaluated lysosomal degradation activity by confocal microscopy using DQ-BSA, whose cleavage by proteases in an acidic compartment generates a fluorescent product whose fluorescence is dependent on lysosomal activity [57,58]. Interestingly, DQ-BSA fluorescence is significantly reduced in silenced cells, indicating that peripherin is important for lysosomal degradation (Figure 2D).

To confirm the alteration in lysosomal activity in peripherin-silenced cells, we looked at EGFR (epidermal growth factor receptor) degradation using a Western blot analysis. This receptor is internalized in the presence of EGF, and then it is degraded in lysosomes [59,60]. Peripherin silencing strongly reduced EGFR degradation, further confirming lysosomal activity impairment (Figure 2E).

**Figure 3:**
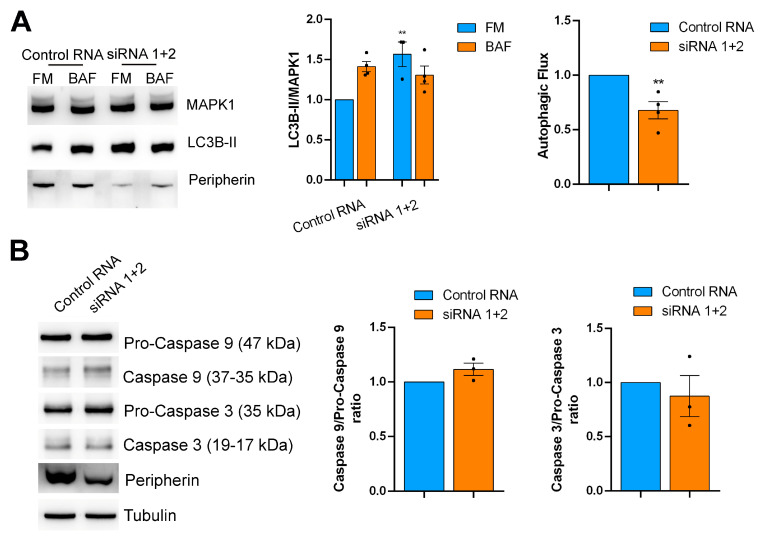
Peripherin silencing affects the autophagic flux but not apoptosis. (**A**) After 96 h, peripherin-silenced or control Neuro2a cells, as indicated, were incubated with full medium (FM) or full medium supplemented with 100 nM bafilomycin A1 (BAF) for 3 h. Lysates were subjected to a Western blot analysis using antibodies against LC3B, peripherin, and MAPK1 as the loading control. LC3B-II abundance was quantified by a densitometric analysis normalizing against MAPK1. Autophagic flux was calculated as the ratio between normalized LC3B-II in bafilomycin A1-treated and full-medium samples. (**B**) Lysates of the control or peripherin-silenced Neuro2a cells were subjected to a Western blot analysis using antibodies against caspase 9, caspase 3, peripherin, or Tubulin as the loading control. The cleavage of caspases was calculated as the ratio between cleaved caspase and pro-caspase. ** = *p* < 0.01.

To confirm that the reduced fluorescence of DQ-BSA and EGFR degradation were due to altered lysosomal activity and not to a general impairment of endocytosis, we looked at RAB proteins involved in different steps of endocytosis compared to RAB7A. Indeed, we analyzed the levels of RAB4 (involved in recycling from early endosomes), RAB5 (involved in the early steps of endocytosis), RAB9 (involved in the transport from early endosomes to the trans-Golgi network), and RAB11 (involved in the recycling of internalized proteins from recycling endosomes to the plasma membrane) [61]. Unlike RAB7A, none of them were affected by peripherin silencing, suggesting that peripherin acts only on the late endocytic steps controlled by RAB7A. Among the ALP markers analyzed, we found that Beclin 1 was more abundant in peripherin-silenced cells, suggesting autophagosome accumulation. Therefore, we decided to investigate the autophagic flux in peripherin-silenced cells using Bafilomycin A1, which is a V-ATPase inhibitor used to prevent lysosomal turnover of the autophagosome content [62]. Notably, the expression of the autophagosome marker LC3B-II (microtubule associated protein 1 light chain 3B) [63] was higher in the silenced cells compared to the control cells both grown in full medium, and consequently, the autophagic flux, measured by looking at the ratio of normalized LC3B-II levels between bafilomycin A1 and full medium of the same sample, was strongly inhibited when peripherin was silenced, suggesting a role of this IF protein in autophagosome–lysosome fusion (Figure 3A).

To better understand whether autophagic flux inhibition was dependent on a direct effect of peripherin silencing on autophagy and not caused by an off-target effect on other pathways, such as apoptosis, we looked at caspase 9 and 3 cleavage, respectively, the initiator and the effector caspases in this process [64]. We demonstrated that peripherin silencing did not affect caspase abundance or cleavage, therefore having no effect on apoptosis and influencing only the autophagic pathway (Figure 3B).

### 2.3. The Expression of Peripherin in Silenced Cells Rescues TFEB Abundance and Lysosomal Functionality

**Figure 4:**
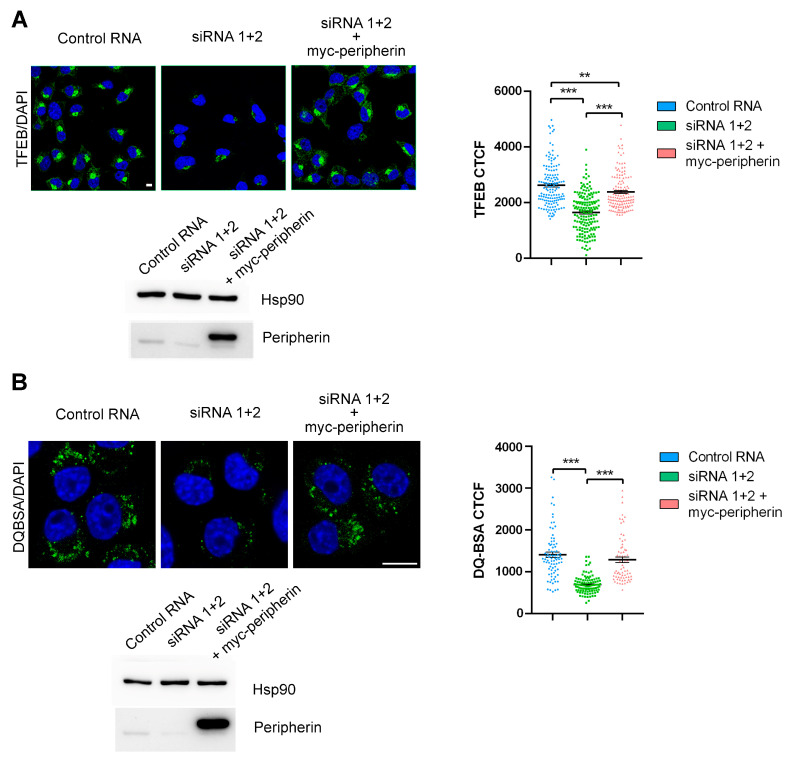
Peripherin expression after silencing rescues TFEB abundance and lysosomal functionality. (**A**) Peripherin-silenced or control Neuro2A cells, after 48 h, were transfected with a plasmid encoding myc-tagged peripherin, and after another 48 h, they were fixed, permeabilized, and immunolabeled using an anti-TFEB antibody followed by an Alexa488-conjugated secondary antibody. Nuclei were stained with DAPI. Bar = 10 µM. TFEB CTCF was calculated using ImageJ. To check transfection, a Western blot analysis was performed using anti-peripherin and anti-Hsp90 antibodies, with the latter being used as a loading control. (**B**) Peripherin-silenced or control Neuro2A cells after 48 h were transfected with a plasmid encoding myc-tagged peripherin, and after another 48 h, they were treated with DQ-Green BSA for 8 h and then fixed. Nuclei were stained with DAPI. Bar = 10 µM. DQ-BSA CTCF was calculated using ImageJ. To check transfection, a Western blot analysis was performed using anti-peripherin and anti-Hsp90 antibodies, with the latter being used as a loading control. ** = *p* < 0.01; *** = *p* < 0.001.

To demonstrate that some of the observed altered phenotypes were unequivocally due to peripherin silencing, we transfected silenced cells with a plasmid coding for myc-tagged peripherin. Interestingly, the re-expression of peripherin was able to rescue TFEB abundance and DQ-BSA fluorescence, indicating that the phenotypes observed following peripherin silencing were not due to off-target effects and were specifically due to peripherin silencing, indicating that peripherin has an important role in lysosomal biogenesis and functionality (Figure 4A,B).

**Figure 5:**
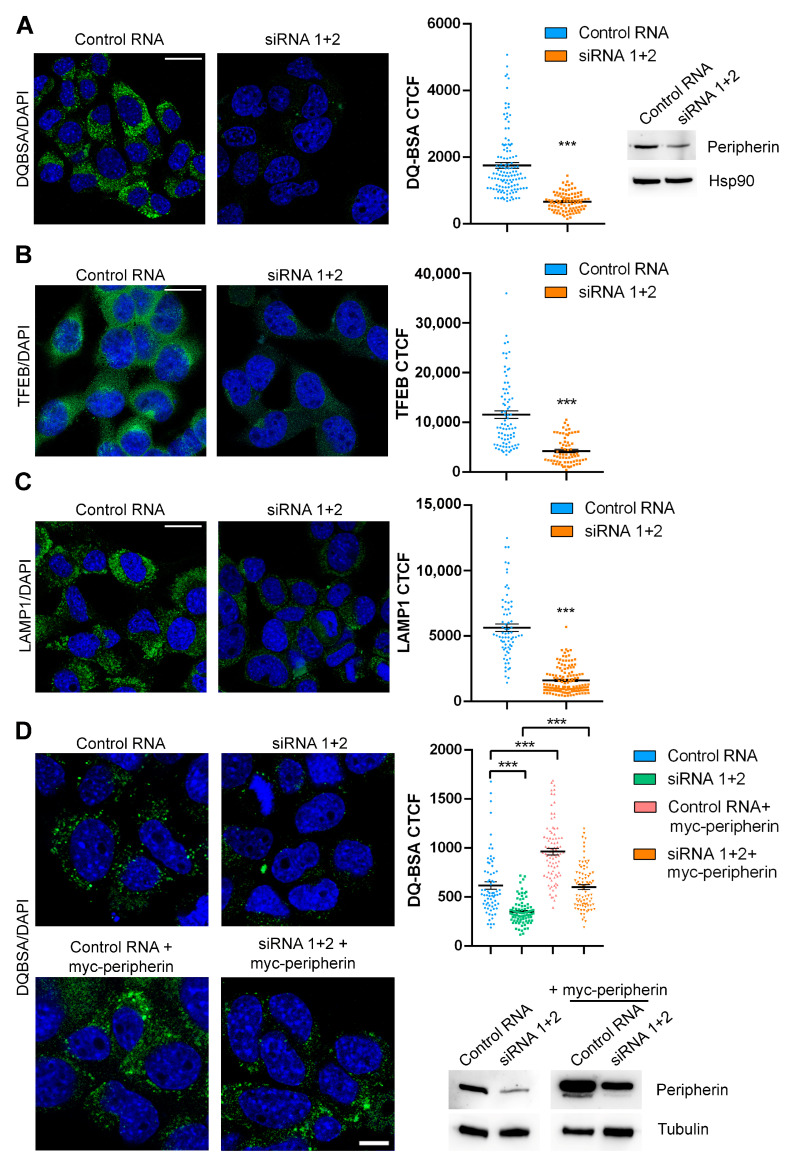
Peripherin silencing affects lysosomal biogenesis and functionality in NSC34 cells. (**A**) NSC34 cells were treated with peripherin siRNA or with a control RNA for 96 h, incubated with DQ-Green BSA for 8 h, and then fixed. Nuclei were stained with DAPI. Bar = 10 µM. DQ-BSA CTCF was calculated using ImageJ. To control silencing, a Western blot analysis was performed using antibodies against peripherin, and Hsp90 was used as the loading control. (**B**,**C**) NSC34 cells were treated with peripherin siRNAs or with a control RNA for 96 h, fixed, permeabilized, and immunolabeled with anti-TFEB (**B**) or anti-LAMP1 (**C**) antibodies followed by an Alexa488-conjugated secondary antibody. Nuclei were stained with DAPI. Bar = 10 µM. ImageJ software was used for the CTCF calculation. (**D**) NSC34 cells were treated with peripherin siRNA or with a control RNA for 48 h and then transfected using a plasmid coding for myc-peripherin. After 48 h, cells were lysed, and samples were subjected to a Western blot analysis to check silencing and transfection or treated with DQ-BSA. Nuclei were stained with DAPI. Bar = 10 µM. DQ-BSA CTCF was calculated using ImageJ. *** = *p* < 0.001.

To confirm these data in other cells, we used the NSC34 cell line, an ibridoma cell line obtained from the fusion of neuroblastoma and spinal cord motor neuron cells. We demonstrated that the DQ-BSA fluorescence intensity was strongly reduced in this cellular model upon peripherin silencing, confirming the important role of peripherin in lysosomal activity (Figure 5A).

Moreover, we also demonstrated that peripherin silencing strongly reduced the intensity of both TFEB and LAMP1 by immunofluorescence experiments, similarly to what we demonstrated in Neuro2A cells (Figure 5B,C).

To evaluate the effect of the overexpression of peripherin in control or silenced cells, we treated NSC34 cells with control RNA or siRNAs against peripherin, and then we transfected them with a plasmid encoding myc-peripherin. We confirmed that peripherin silencing was associated with a decrease in lysosomal degradative activity, as demonstrated by DQ-BSA reduced fluorescence in silenced cells. Moreover, the re-expression of peripherin in silenced cells was able to rescue DQ-BSA fluorescence and, therefore, lysosomal activity, demonstrating that the effect was specifically due to peripherin silencing. Finally, the overexpression of peripherin in control cells led to an increase in lysosomal degradative activity (Figure 5D).

These data confirm the importance of peripherin in lysosomal biogenesis and functionality.

### 2.4. The Overexpression of Peripherin Influences TFEB Abundance but Not the Autophagic Flux

**Figure 6:**
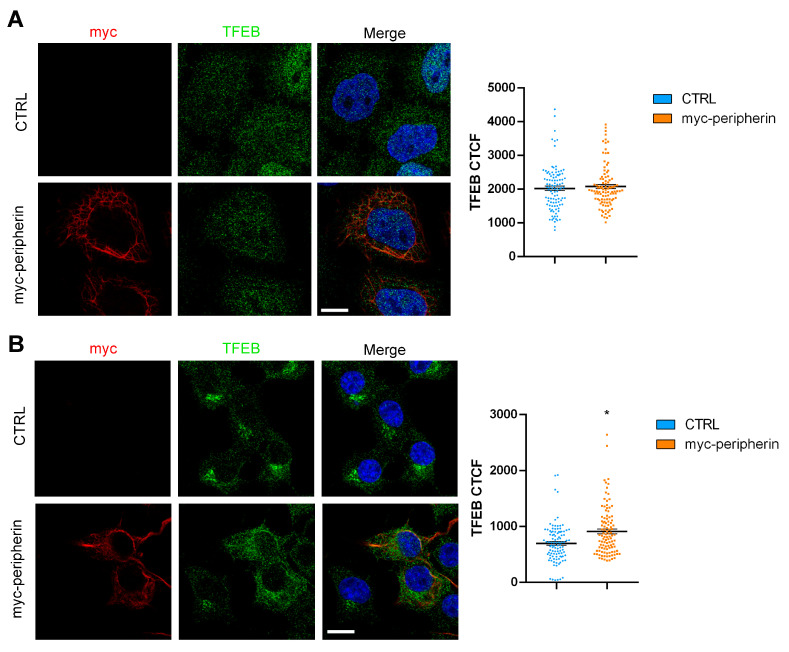
Peripherin overexpression is associated with an increased abundance of TFEB in Neuro2a cells. HeLa cells (**A**) or Neuro2a cells (**B**) were transfected with a plasmid coding for myc-peripherin for 48 h, and then they were fixed and immunolabeled using antibodies against myc and TFEB followed by Alexa568- and Alexa488-conjugated secondary antibodies, while the nuclei were stained with DAPI. Bar = 10 µM. TFEB CTCF was calculated using ImageJ software. * = *p* < 0.05.

**Figure 7:**
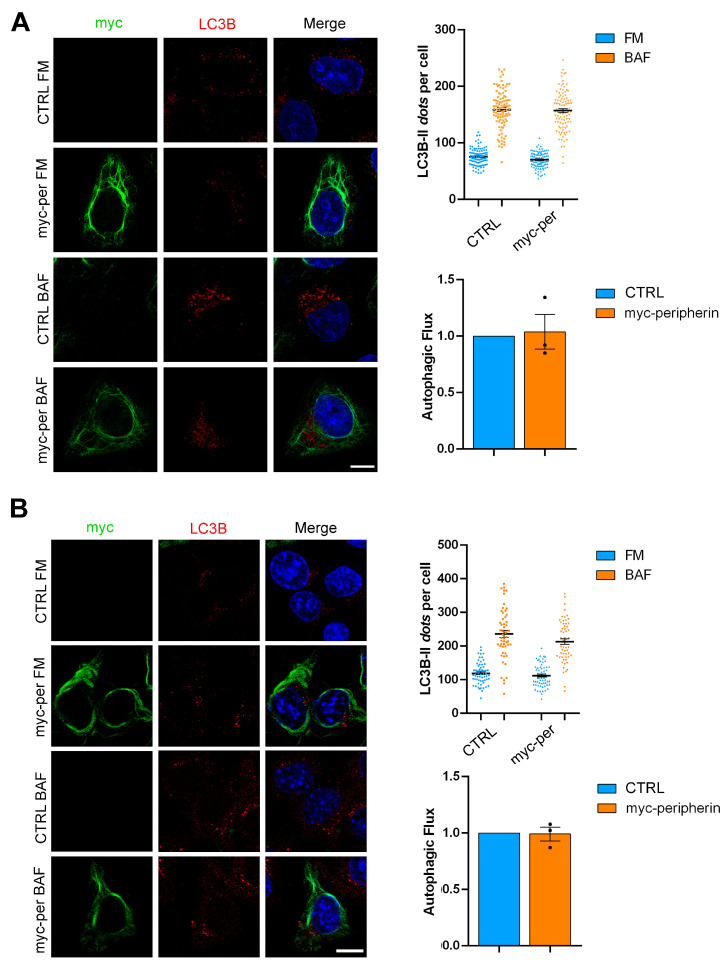
Peripherin overexpression does not affect the autophagic flux. HeLa cells (**A**) or Neuro2a cells (**B**) were transfected with plasmid coding for myc-peripherin for 48 h. Cells were then treated with 100 nM bafilomycin A1 for three hours and then fixed and immunolabeled using antibodies against myc and LC3B, followed by Alexa488- and Alexa568-conjugated secondary antibodies, while the nuclei were stained with DAPI. Bar = 10 µM. LC3B dots were calculated using ImageJ software. The autophagic flux was calculated as the ratio between LC3B-II dots in bafilomycin A1-treated and full-medium samples.

To evaluate the effect of peripherin expression, we decided to transfect HeLa cells (which represent a model of peripherin-null cells since they do not express peripherin) and Neuro2a cells in order to evaluate the effects of peripherin overexpression. In these cellular models, we evaluated TFEB abundance and the autophagic flux. We observed that in HeLa cells, TFEB abundance and autophagic flux were unchanged following peripherin expression (Figure 6A and Figure 7A). In contrast, the overexpression of peripherin in Neuro2a cells caused an increase in the abundance of TFEB (Figure 7A), although the autophagic flux was not affected (Figure 7B).

These data, together with the data obtained in NSC34 cells in which the overexpression of peripherin was associated with an increase in lysosomal degradative activity, further confirm the positive effect of peripherin on the late endocytic steps.

## 3. Discussion

IFs have an important role in organelle position, shape, and functions interacting with components of vesicular transport machinery or with proteins controlling organelle functions [65]. At present, few IF proteins have been related to endocytic organelle positioning and functionality. For instance, it has been demonstrated that vimentin regulates endocytosis and endosome motility in astrocytes [25,26]. In addition to vimentin, GFAP and desmin also regulate lysosomal motility and positioning [66]. The control of endocytosis by vimentin filaments may be due to vimentin interaction with RAB5A, involved in the early steps of endocytosis, with RAB4A, which regulate recycling from endosomes to the plasma membrane, and with RAB7A, which is important for transport to late endosomes and lysosomes [67,68,69,70,71]. In addition, vimentin seems to be connected to the degradative pathway by interacting with lysosomes and autophagosomes [23]. Indeed, vimentin interacts with the adaptor protein AP-3, which is involved in endosomal-to-lysosomal transport and in the control of lysosomal pH and zinc uptake [23]. Interestingly, vimentin shares some of its interactors with another class III IF protein, peripherin. Indeed, it has been demonstrated that peripherin also interacts with AP-3 and RAB7A [23,34]. These common interactions suggest that peripherin could also be involved in vesicular trafficking and endocytic organelle positioning and functioning even though a direct demonstration is missing.

In order to establish the role of peripherin in the endocytic degradative pathway, we silenced peripherin using two siRNAs and, first of all, we evaluated the effect of its silencing on LAMP1 abundance and lysosomal positioning. We demonstrated that peripherin silencing affects LAMP1 expression and lysosomal positioning. Indeed, while late endosomes and lysosomes were localized mainly in the perinuclear area in the control cells, they were spread in the entire cytoplasm when peripherin was silenced. To confirm that the observed alteration was due to silencing, we re-expressed peripherin in silenced cells and we observed that LAMP1 abundance and intracellular distribution were rescued, demonstrating that this phenotype was due to peripherin silencing and not to the off-target effects of siRNAs. Interestingly, a previous study showed that the inhibition of vimentin by Withaferin A caused the juxtanuclear localization of lysosomes [72]. Therefore, our data highlight a different role of peripherin in regulating lysosomal positioning compared to vimentin since its silencing determined the dispersion of lysosomes, while vimentin inhibition caused the accumulation of lysosomes in the perinuclear area. This suggests that the two IF proteins could both regulate lysosomal positioning but in opposite directions. Moreover, our data about reduced LAMP1 abundance in peripherin-depleted cells is consistent with the decreased LAMP2 abundance previously observed in vimentin-deficient cells. Indeed, these cells showed reduced levels of LAMP2 but not of early endosomal proteins as the transferrin receptor. Therefore, vimentin and possibly also peripherin, both interacting with AP-3, could selectively regulate the trafficking of the lysosomal cargoes LAMP proteins [23]. We also analyzed other proteins involved in ALP using a Western blot analysis, and we demonstrated that in addition to LAMP1, RILP, V1G1, and RAB7A are also downregulated in peripherin-depleted cells, suggesting a serious impairment of the late endocytic traffic. To better evaluate this aspect, we looked at TFEB abundance. Indeed, the reduced abundance of LAMP1 suggests that peripherin-depleted cells possess fewer lysosomes, and the transcription factor TFEB is the master regulator of lysosomal biogenesis [50,73]. Interestingly, upon peripherin silencing, the levels of TFEB were drastically reduced, as demonstrated by immunofluorescence and Western blot experiments. Therefore, peripherin silencing affects lysosomal biogenesis, impairing TFEB abundance, and reduces the number of lysosomes, explaining the diminished levels of LAMP1. Interestingly, another master regulator of lysosomal biogenesis, TFE3, was downregulated in peripherin-depleted cells. We can speculate that the observed downregulation of these transcription factors is not directly dependent on peripherin silencing, but it could be mediated by other factors influenced by peripherin, such as RAB7A. In peripherin-depleted cells, we demonstrated that RAB7A levels were decreased. In a recent study, we showed that cells resistant to cisplatin showed RAB7A downregulation, and this was associated with decreased levels of PGC-1α (peroxisome proliferator-activated receptor-gamma coactivator 1 alpha) and NRF-1 (nuclear respiratory factor 1), establishing a correlation between these factors [74]. Interestingly, PGC-1α upregulates and activates TFEB, while NRF-1 binds to the promoter of the TFE3 gene and directly regulates TFE3 expression [55,75,76]. Therefore, peripherin silencing causes RAB7A downregulation which, in turn, could lead to reduced levels of NRF-1 and PGC-1α that negatively affect TFEB and TFE3, reducing lysosomal biogenesis.

TFEB regulates a great number of genes, among which is LAMP1, but also the selective autophagy receptor p62/SQSTM1 and several subunits of the vacuolar H^+^-ATPase (V-ATPase), such as ATP6V0D1 [77]. Therefore, the decreased abundance of these proteins that we observed is consistent with TFEB downregulation since the markers that we analyzed are targets of this transcription factor.

Considering that TFEB overexpression increases the number of lysosomes and the levels of lysosomal enzymes enhancing lysosomal degradative activity [78], and considering that we observed, on the contrary, that TFEB is downregulated in peripherin-depleted cells, we decided to evaluate lysosomal activity using a DQ-BSA assay. As expected, peripherin silencing strongly affects DQ-BSA fluorescence and, therefore, lysosomal degradative capability.

To confirm the importance of peripherin in lysosomal activity, we evaluated the degradation of EGFR. The inhibited degradation of EGFR suggests the impairment of lysosomal degradation or impaired transport of the receptor to lysosomes [79,80]. Notably, the amount of degraded EGFR was significantly reduced in peripherin-depleted cells, further demonstrating the malfunctioning of the late endocytic pathway upon peripherin silencing. Considering also the data obtained with the DQ-BSA assay, these data highlight the importance of the peripherin–RAB7A interaction for proper lysosomal activity. In addition, we previously demonstrated that RAB7A silencing inhibited EGFR degradation [81], and we obtained similar results upon peripherin silencing, which also caused the downregulation of RILP, a RAB7A effector which controls the transport of endosomes from the periphery to the perinuclear region during endosome maturation [82]. Therefore, peripherin silencing could affect endosome maturation and lysosomal activity, downregulating RAB7A and RILP.

A common feature of vimentin and peripherin-depleted cells is represented by defective lysosomal activity. Indeed, we demonstrated that the fluorescence of the DQ-BSA probe was decreased by peripherin silencing. Similarly, it was previously demonstrated that vimentin silencing impaired the acidification of endocytic compartments, thus affecting lysosomal activity [23]. Moreover, we demonstrated that peripherin silencing was associated with the downregulation of the subunits of the V-ATPases ATP6V1G1 and ATP6V0D1 and to the reduced levels of RILP, which is fundamental for the assembly of V-ATPase on endosomes through its interaction with ATP6V1G1 [45]. Therefore, the IF proteins vimentin and peripherin could both play a role in the acidification of endocytic organelles. Among the markers analyzed, Beclin 1 was upregulated in peripherin-depleted cells. Beclin 1 is an important player in autophagy [83,84], and its upregulation coupled with compromised lysosomal activity suggests that the autophagy pathway may not work properly in peripherin-depleted cells. As expected, peripherin silencing determined an increase in the abundance of LC3B-II and the inhibition of the autophagic flux, indicating a role of peripherin in autophagosome maturation and, therefore, in autophagy degradation. It was previously demonstrated that TFEB overexpression enhances the degradation of bulk autophagy substrates [85]. Considering that we observed reduced TFEB abundance following peripherin silencing, it is not surprising that autophagy was impaired. Moreover, a recent report showed that vimentin inhibition caused the accumulation of autophagosomes and interfered with autophagosome–lysosome fusion [72]. Therefore, similarly to vimentin, peripherin has a role not only in endosome maturation but also in autophagosome maturation, being important for the endocytic and autophagy pathways.

Therefore, based on our data and on the previous literature, we can speculate similar roles for vimentin and peripherin in regulating lysosomal acidification, autophagosome and endosome maturation, and the trafficking of lysosomal cargoes. However, while vimentin silencing caused lysosomal accumulation in the perinuclear area, peripherin silencing led to the spreading of lysosomes in the cytoplasm, suggesting the involvement of both of these proteins in lysosomal movement but in opposite directions. Moreover, we demonstrated that peripherin overexpression did not alter the autophagic pathway, but it was associated with increased TFEB abundance and lysosomal degradative activity, further confirming its importance in the late steps of endocytosis probably through its interaction with RAB7A.

Interestingly, peripherin is the more expressed IF protein in PC12 cells, a cellular model widely used to study neurite outgrowth [33]. Therefore, as peripherin is mainly expressed in neurons of the PNS, we can speculate that, in these neurons, peripherin could have a key role in lysosomal activity and autophagy compared to other IFs.

In conclusion, we demonstrated that peripherin regulates lysosomal abundance, positioning, and degradative activity, affecting both the endocytic and autophagic pathways. Indeed, it affects the abundance of the master regulator of lysosomal biogenesis TFEB. Since peripherin is expressed in the neurons of the PNS, the modulation of peripherin amount or function could promote the onset of neurodegenerative diseases, many of which are characterized by alterations in the endocytic and autophagy pathways. Moreover, it is worth emphasizing that RAB7A also plays specific roles in neurons such as the control of neurotrophin axonal retrograde transport and signaling, neurite outgrowth, and the final phase of immature cortical neuron migration [86,87,88], while peripherin has a role in axonal regeneration [89,90]. Therefore, an abnormal interaction between these two proteins could cause neurodegeneration.

## 4. Materials and Methods

### 4.1. Antibodies

Primary antibodies used in this study were goat polyclonal anti-peripherin (sc-7604, 1:500), mouse monoclonal anti-GAPDH (sc-25778, 1:2000), mouse monoclonal anti-RAB7A (sc-376362, 1:500), goat polyclonal anti-beclin 1 (sc-10086; 1:200), mouse monoclonal anti-Hsp90 (sc-13119, 1:5000), mouse monoclonal anti-MAPK1 (sc-514302; 1:500), mouse monoclonal anti-ATP6V0D1 (sc-81887, 1:100), mouse monoclonal anti-myc (sc-40, 1:50 for IF), rabbit polyclonal anti-myc (sc-789, 1:50 for IF), and mouse monoclonal anti-β-actin (sc-47778, 1:5000), which were obtained from Santa Cruz Biotechnology (Santa Cruz, CA, USA); rabbit polyclonal anti-TFE3 (HPA023881; 1:1000) obtained from ThermoFisher Scientific (Waltham, MS, USA); rabbit polyclonal anti-LAMP1 (ab24170, 1:4000), mouse monoclonal anti-V1G1 (sc25333, 1:100), and rabbit polyclonal anti-RILP (ab140188, 1:500) from Abcam (Cambridge, UK); rabbit polyclonal anti-peripherin (AB1530, 1:100 for IF) from Merck Millipore (Burlington, MS, USA); sheep polyclonal anti-EGFR (20-ES04, 1:1000) from Fitzgerald (North Acton, MS, USA); mouse monoclonal anti-LC3B (0231-1000, 1:500 for WB, 1:200 for IF) from Nanotools (Teningen, Germany); rabbit polyclonal anti-TFEB (A303-673A, 1:400) from Bethyl Laboratories (Montgomery, TX, USA); mouse monoclonal anti-LAMP1 (1D4B, 1:50 for IF) from the Developmental Studies Hybridoma Bank (Iowa City, IA, USA); rabbit polyclonal anti-caspase 3 (9662, 1:1000), mouse monoclonal anti-caspase 9 (9508, 1:1000), rabbit polyclonal anti-RAB4 (2167, 1:1000), rabbit polyclonal anti-RAB5 (3547, 1:1000), rabbit polyclonal anti-RAB9 (5118, 1:1000), and rabbit polyclonal anti-RAB11 (5589, 1:2000) from Cell Signaling Technology (Danvers, MA, USA); and mouse monoclonal anti-SQSTM1/p62 (1:1000; 610833) from BD Biosciences (Franklin Lakes, NJ, USA). Secondary antibodies conjugated to fluorochromes (used at 1:400 dilution) or HRP (used at 1:5000 dilution) were from Invitrogen (Carlsbad, CA, USA), Fitzgerald, or Bio-Rad (Hercules, CA, USA).

### 4.2. Cell Culture

Neuro2a cells (RRID:CVCL_0470) were mouse neuroblasts with neuronal and ameboid stem cell morphology isolated from brain tissue [91], and they were purchased from ATCC. NSC-34 cells were mouse motor neurons (RRID:CVCL_D356) derived from the fusion of motor neuron-enriched embryonic day 12–14 spinal cord cells with the aminopterin-sensitive neuroblastoma N18TG2 cell line [92], and they were kindly provided by Prof. Angelo Poletti (Università degli Studi di Milano, Milan, Italy). HeLa cells (CVCL_0030) were derived from human papillomavirus-related cervical adenocarcinoma. All cell lines were cultured in Dulbecco’s modified Eagle’s medium (DMEM) supplemented with 10% fetal bovine serum (FBS), 2 mM L-glutamine, 100 U/mL penicillin, and 10 mg/mL streptomycin in a 5% CO_2_ incubator at 37 °C. Cells were periodically checked from mycoplasma infections. The reagents for cell culture were from Euroclone (Pero, MI, Italy).

### 4.3. Silencing and Transfection

Silencing experiments were performed using Metafectene SI^+^ from Biontex (Martinsried, Germany) following the manufacturer’s instructions. Cells were analyzed 96 h after transfection. Small interfering RNAs (siRNAs) were purchased from MWG-Biotech (Ebersberg, Germany).

The sequences of siRNAs used in this study are listed below:

Control RNA, as negative control (sense sequence 5′-ACUUCGAGCGUGCAUGGCUTT-3′ and antisense sequence 5′-AGCCAUGCACGCUCGAAGUTT-3′);

Mouse peripherin siRNA 1 (sense sequence 5′-GCAAGAUUGAGUCUCUGAUTT-3′ and antisense sequence 5′-AUCAGAGACUCAAUCUUGCTT-3′);

Mouse peripherin siRNA 2 (sense sequence 5′-GCAGAGGUUAGAAGAAGAATT-3′ and antisense sequence 5′-UUCUUCUUCUAACCUCUGCTT-3′).

In rescue experiments, 48 h after silencing, cells were transfected with a plasmid coding for myc-tagged peripherin purchased from Origene (RC207561, Rockville, MD, USA) and using Metafectene Pro from Biontex following the manufacturer’s instructions. Cells were analyzed 48 h after transfection.

Silencing and rescue experiments were performed on cells seeded in a 6-well plate. In each well, coverslips were previously placed. At the end of silencing or transfection time, coverslips were moved in a 24-well plate in order to be immunostained, and the remaining cells in the 6-well plate were lysed to control peripherin silencing or overexpression of the exogenous protein.

### 4.4. Immunofluorescence

Cells were grown on 11 mm coverslips and then fixed with 3% paraformaldehyde for 20 min at room temperature. Permeabilization was performed using 0.1% Triton X-100 in PBS for 10 min, and then cells were incubated with primary and secondary antibodies both diluted in 0.1% saponin in PBS for 40 min. Nuclei were stained with DAPI (4′,6-Diamidino-2-Phenylindole). Coverslips were mounted and viewed with Zeiss LSM 700 confocal microscope. For LC3B immunostaining, cells were fixed and permeabilized with methanol at −20 °C for 5 min. Corrected Total Cell Fluorescence (CTCF) was calculated as previously described [93,94], analyzing only cells showing a clear downregulation of peripherin when immunostaining was performed or analyzing at least 100 cells for experiment, averaging the obtained results and checking peripherin silencing using a Western blot analysis. To evaluate lysosomal distribution, cells showing a clear lysosomal cluster in the perinuclear area were manually counted and considered as cells with compact lysosomes, while cells not showing a clear lysosomal cluster were counted as cells with dispersed lysosomes. LC3B dots were measured with the tool “Analyze Particle” of ImageJ, as previously described [58].

### 4.5. Western Blotting

Cells were lysed in Laemmli buffer [100 mM Tris–HCl, pH 6.8, 4% (*w*/*v*) SDS, 0.2% (*w*/*v*) bromophenol blue, 20% glycerol, and 200 mM DTT (dithiothreitol)] or in MAPK lysis buffer (20 mM HEPES pH 7.5, 10 mM EGTA, 40 mM beta-glycerophosphate, 1% NP-40, 2.5 mM MgCl_2_, 2 mM NaF, 1 mM DTT, and Roche protease inhibitor cocktail (05056489001, Roche Diagnostics, Monza, Italy) and subjected to a Western blot analysis as previously described [95,96]. Briefly, after SDS-PAGE, proteins were transferred on PVDF (polyvinylidene) membranes (IPVH00010, Merck-Millipore, Burlington, MA, USA). After being incubated in blocking solution (5% milk in PBS) for 1 h, membranes were incubated with primary antibodies overnight at 4 °C and HRP-conjugated secondary antibodies for 1 h at room temperature. Clarity or Clarity max reagents from Bio-Rad were used for the chemiluminescence reaction, and the signal was captured using ChemiDoc MP Imaging Systems (Bio-Rad, Hercules, CA, USA). Densitometric analysis was performed using Image Lab software 6.1 (Bio-Rad).

### 4.6. DQ-BSA Assay

Cells seeded on 11 mm glass coverslips were treated with 10 µg/mL DQ-Green BSA (D12050, ThermoFisher Scientific, Waltham, MS, USA) or DQ-Red BSA (D12051, ThermoFisher Scientific) for 8 h. Then, cells were fixed with 3% paraformaldehyde for 20 min and immunolabeled as previously described. Coverslips were mounted with Mowiol, and images were acquired with LSM700 confocal laser scanning microscope provided with a 63X objective (Zeiss, Jena, Germany). Corrected Total Cell Fluorescence (CTCF) was calculated as previously described [93,94], analyzing only cells showing a clear downregulation of peripherin when it was immunostained or analyzing at least 100 cells for experiment, averaging the obtained results and checking peripherin silencing using a Western blot analysis.

### 4.7. EGFR (Epidermal Growth Factor Receptor) Degradation Assay

Cells were treated with 10 μg/mL cycloheximide in serum- and antibiotic-free medium for 1 h at 37 °C, then stimulated with 100 ng/mL EGF for 15 min and 3 h and lysed with RIPA buffer (50 mM Tris–HCl, pH 8.0, with 150 mM sodium chloride, 1.0% Igepal CA-630 (NP-40), 0.5% sodium deoxycholate, and 0.1% sodium dodecyl sulfate) plus protease inhibitor cocktail (Roche, Mannheim, Germany). The levels of degraded EGFR were determined by Western blotting.

### 4.8. Statistical Analysis

Experiments were performed at least in triplicate. For confocal microscopy experiments, at least 50 cells per experiment were considered, and Corrected Total Cell Fluorescence (CTCF) was calculated as previously described [93,94]. Graphs represent the mean of at least three independent experiments ± standard error mean. Statistical analysis was performed using Student’s *t* test or one-way ANOVA followed by Dunnett’s test for multiple comparisons (* = *p*  <  0.05, ** = *p*  <  0.01, and *** = *p*  <  0.001).

## Data Availability

The original contributions presented in this study are included in the article. Further inquiries can be directed to the authors.
